# Psychosocial hazards and work-life balance: the role of workplace conflict, rivalry, and harassment in Latvia

**DOI:** 10.3389/fpsyg.2025.1494288

**Published:** 2025-02-20

**Authors:** Diāna Inga Paegle, Svetlana Lakiša, Linda Matisāne, Monta Matisāne, Linda Paegle, Kristīne Mārtinsone, Daiga Kamerāde, Valentīna Krūmiņa, Elīna Akmane, Amanda Ķule, Ivars Vanadziņš

**Affiliations:** ^1^Institute of Occupational Safety and Environmental Health, Rīga Stradiņš University, Riga, Latvia; ^2^Department of Health Psychology and Pedagogy, Rīga Stradiņš University, Riga, Latvia; ^3^School of Health and Society, University of Salford, Salford, United Kingdom

**Keywords:** work-life balance, psychosocial hazards, workplace conflicts, sexual harassment, workplace violence, rivalry, work characteristics

## Abstract

**Background:**

Even though the link between the psychosocial work environment and work-life balance (WLB) has been thoroughly researched, there is limited evidence evaluating the impact of workplace violence, sexual harassment, conflicts, and rivalry on WLB.

**Methods:**

A cross-sectional study was conducted among 2,471 respondents in Latvia from December 20, 2021, to July 14, 2022. WLB was measured through a survey question assessing the frequency of work-life imbalance, with responses categorized into dichotomous variables. The study evaluated the association between the selected workplace psychosocial hazards (conflicts, rivalry, psychological abuse, physical abuse, and sexual harassment), work characteristics, socio-demographic factors, and WLB by using binomial logistic regression.

**Results:**

Our study reveals a significant lack of WLB among Latvian employees. A striking one-third of the respondents (30.9%, *n* = 762) reported experiencing this imbalance. The odds of WLB decrease with age, with the youngest age group having twice the odds compared to the oldest age group. Lower education levels and lower income groups also show significantly lower odds of WLB. Notably, those who have experienced selected workplace psychosocial hazards, such as sexual harassment or psychological abuse, have five- and three-times higher odds of work-life imbalance (aOR = 4.90 with 95% CI 2.06–11.67 and aOR = 3.47 with 95% CI 2.75–4.35, respectively). All types of conflicts at work significantly increase the odds of a lack of WLB. Our findings also indicate that WLB varies depending on various work characteristics, such as job position, work sector, company size, length of service, and remote or on-site work.

**Conclusion:**

Our study highlights the importance of addressing WLB in the context of workplace conflicts, rivalry, violence, and harassment. It provides indirect evidence favoring leadership quality and manager training instead of employee training in diminishing psychosocial hazards. Practical implications include prioritizing leadership development programs focusing on conflict resolution and fostering a supportive organizational culture to improve employee WLB.

## Graphical abstract

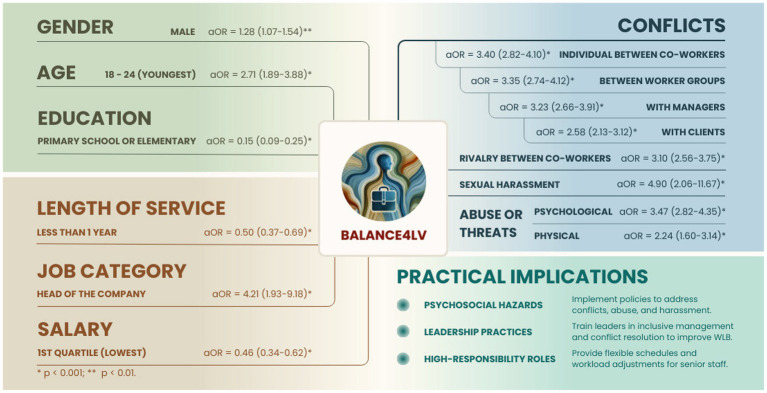

## 1 Introduction

WLB or work-life integration is a concept that emerged in the 1960s with the arrival of Generation X, who was the first to favor it as a boomerang effect to the work-accentuated previous generation; WLB speaks to creating the most compatible work and life for employees (Brough et al., 2020; Kalliath & Brough, 2008; Ramachandran, 2020). The reason for focusing attention on employees is the intensifying competitive pressure through the twenty-first century (Hitt et al., 1998). It makes employees the only sustainable source of competitive advantage and the return on investment in organizations (Wong et al., 2020; Luthans & Youssef, 2004). On the other hand, employees are increasingly and consistently trying to put their WLB first (Drnovšek et al., 2024). They do so despite being connected and engaged in their current roles, and such a choice is even more pronounced within the younger generation of employees (Picton, 2021). The employee preference of WLB is reflected in the priorities regulatory agencies set globally (European Commission, 2021; The European Parliament and the Council of the European Union, 2019; Kelliher et al., 2019). Also, organizations that put employee WLB first have better business results, including higher profits (Naylor, 2004). Thus, achieving a win-win status for employees and organizations means investing in employees as the primary asset of organizations (Kelliher et al., 2019).

Furthermore, such investment should help employees perceive a positive WLB, i.e., that their work and non-work activities are compatible and promote growth according to an individual’s life priorities (Gragnano et al., 2020). It is a preferred definition of WLB in a field riddled with disagreements on how to define WLB (European Agency for Safety and Health at Work, 2012). Even though WLB is a concept central to organizational productivity, return on investment, employee retention, and employee happiness, WLB is one of the least studied phenomena academically (Haar et al., 2014; Guest, 2002). Consequently, WLB has become “a concept whose popular usage has outplaced its theoretical development” despite WLB being entered into the European Pillars of Social Rights (Brough et al., 2020; Haar et al., 2014; Beauregard & Henry, 2009; European Agency for Safety and Health at Work, 2019).

Moreover, the existing academic literature on WLB is unbalanced, as it often takes work-family balance as a proxy for WLB in most studies (Fan & Smith, 2017; Lingard & Francis, 2009). For example, 74% of qualitative and 91% of quantitative studies on WLB from 1987 to 2006 focused on work-family balance (Kelliher et al., 2019; Guest, 2002; Chang et al., 2010). Over the past 15 years, research has further explored the cross-pollination of work and family life within the context of WLB (Keeney et al., 2013; Sirgy & Lee, 2018; Casper et al., 2017; Verma et al., 2024). However, emerging evidence suggests that employees (irrespective of their age) increasingly prioritize the work-health component of WLB over the work-family balance (Gragnano et al., 2020).

This shift in focus aligns with the growing recognition of workplace psychosocial hazards as critical determinants of employee health and well-being. Since the 1970s, such hazards—including workplace conflicts, violence, and harassment—have been identified as pivotal to the psychosocial work environment (Kalimo et al., 1987; Guerrero-Barona et al., 2020; Rugulies, 2019). Consequently, regulators have prioritized the prevention and management of these hazards to support both individual well-being and organizational success (The European Parliament and the Council of the European Union, 2019; Gragnano et al., 2020; Eurofound, 2024; Llave & Weber, 2020; European Agency for Safety and Health at Work, 2024; Lama, 2018; Gualano et al., 2023). Furthermore, the COVID-19 pandemic has amplified the need for minimizing psychosocial risks, as the shift to remote and hybrid work has fundamentally altered the organization of work (Eurofound, 2024; Llave et al., 2023).

The COVID-19 pandemic has profoundly reshaped workplace dynamics, accelerating the adoption of remote and hybrid work arrangements. These shifts have brought unique challenges to employees, such as blurred boundaries between work and personal life, increased workloads, reduced social interaction, and limited access to in-person support networks. Such challenges have exacerbated existing workplace psychosocial risks and introduced new stressors, such as digital overload, isolation, and difficulties in managing a healthy WLB (Eurofound, 2024; Gualano et al., 2023; Llave et al., 2023).

Remote work, while offering flexibility, has also led to an “always-on” culture, where employees feel compelled to be available outside standard working hours (Gualano et al., 2023; Llave et al., 2023). This culture has been linked to heightened stress and diminished personal time, contributing to a negative WLB. For employees in hybrid setups, navigating shifting expectations between in-office and remote work has added further complexity to their work-life integration (Eurofound, 2024).

The COVID-19 pandemic also highlighted disparities in employees’ ability to adapt to these changes (Gualano et al., 2023). Factors such as job role, access to digital infrastructure, and individual coping mechanisms have influenced how employees experience and manage these psychosocial risks (Eurofound, 2024; Llave et al., 2023). For many, the pandemic’s disruption has underscored the importance of organizational policies that prioritize mental health, workplace flexibility, and proactive management of psychosocial hazards to maintain WLB (Llave et al., 2023).

In this context, the management of workplace psychosocial hazards has gained renewed significance (Llave & Weber, 2020; European Agency for Safety and Health at Work, 2024). Organizations are increasingly recognizing the need for targeted interventions to mitigate these risks, such as promoting clear boundaries between work and personal life, enhancing virtual collaboration tools, and fostering a culture of trust and support (Eurofound, 2024; Ramkissoon et al., 2019). These measures are essential not only for employee well-being but also for organizational resilience in an evolving work environment (Wong et al., 2020; Luthans & Youssef, 2004; Naylor, 2004).

Despite these efforts, the broader health implications of psychosocial workplace hazards cannot be overlooked. An extensive body of evidence has linked these hazards to severe outcomes such as mortality, cardiovascular disease, mental illness, and musculoskeletal disorders (Taouk et al., 2020). However, a notable gap remains in understanding the association of psychosocial workplace hazards to WLB, as few studies have delved into this critical relationship (Bjärntoft et al., 2020; Yang et al., 2018).

When examining the current multidimensional and holistic models evaluating psychosocial workplace hazards, conflicts in the workplace (including actions that lead to disputes, such as threats, violence, and sexual harassment), quality of leadership, and vertical trust emerge as significant determinants of the psychosocial environment (Ramkissoon et al., 2019; Clausen et al., 2019). Conflicts can hurt employee well-being, leading to outcomes such as mental health issues, sickness presenteeism, or absenteeism (Lakiša et al., 2021; Kuriakose et al., 2019; De Dreu, 2008; Lakiša et al., 2022; Conway et al., 2016). Similarly, the impact of conflicts on knowledge-sharing intentions between colleagues, innovation, creativity, team performance, and trust have been studied (Wang et al., 2019; Han & Harms, 2010). However, conflicts between worker groups, conflicts with managers, and conflicts with co-workers and their association with WLB have been less explored. The potential positive and negative effects of conflicts on the work environment add a layer of complexity to the issue, making it a crucial area for further research.

Even so, conflicts remain one of the most debated workplace psychosocial hazards. On the one hand, conflicts can negatively affect WLB by hindering mutually beneficial solutions essential for both organizational productivity and employee well-being (Moreira et al., 2023; Wang, 2022; Majer et al., 2021; Rahal & van Beest, 2022). Among these conflicts, interpersonal conflicts are closely linked to rivalry, which arises from interest collision (Rahal & van Beest, 2022). Rivalry influences conflict behavior by fostering a preference for maximizing individual gains over cooperative outcomes (Houston et al., 2000). While rivalry can have motivational benefits, such as encouraging innovation and performance, it may also escalate into conflict, particularly when individuals or groups involved are of similar status (Piezunka et al., 2018).

Rivalry can have a significant impact on WLB, as it often leads to increased competition, stress, and tensions in the workplace (Rahal & van Beest, 2022). Employees engaged in rivalrous dynamics may feel compelled to invest more time and energy into work-related activities, often at the expense of their personal and family life (Rahal & van Beest, 2022; Piezunka et al., 2018). Furthermore, rivalry can undermine collaboration and trust among colleagues, leading to a more hostile work environment, which in turn negatively affects employees’ ability to maintain a healthy balance between work and personal lives (Piezunka et al., 2018; Pelled et al., 1999).

Conversely, a lack of conflicts or rivalry in the workplace may suggest an overly passive or disengaged environment where trust and open communication are insufficient to foster idea-sharing and constructive debate (Pelled et al., 1999; Ayub & Jehn, 2014). This duality underscores the complex role rivalry plays in the workplace—it can both motivate employees and disrupt their WLB, depending on how it is managed and perceived (Schabracq & Cooper, 1979).

Despite its importance, the association of WLB with conflicts and rivalry as workplace psychosocial hazards has received limited attention in existing research. Our study, therefore, aims to address this gap by evaluating the extent to which conflicts and rivalry—both—status similar (employees and co-workers; groups of workers) and status dissimilar (employees and managers; employees and clients) influence employees’ WLB. Moreover, we hypothesize that workplace sexual harassment and workplace violence are events that lead to conflict or are essentially conflicts—intra- and inter-personal, and possibly group and organizational-level conflicts. Our study aimed to evaluate how significant these conflicts are to employee WLB.

Within the workplace, sexual harassment retains its distinct prominence despite the European Parliament Directive 2002/73/EC and consistent global efforts by organizations to prevent it (The European Parliament and the Council of the European Union, 2019; Diez-Canseco et al., 2022). Sexual harassment is a form of discrimination and violence (Burn, 2019). Adverse effects of sexual harassment on the well-being of employees (predominantly women) have been demonstrated, and a multitude of international policies exist to eliminate it (Organization IL, 2019; UN Women, 2023; World Health Organization, 2023). However, detailed effects of sexual harassment on mental health variables, productivity, and absenteeism have been evaluated without much attention to how sexual harassment affects WLB of harassed employees (Nielsen & Einarsen, 2012; Vara-Horna et al., 2023).

Similarly, workplace violence—psychological and physical abuse and threats—has been evaluated in terms of how it affects the physical and mental health of employees, their career choices, and organizational performance (Yu et al., 2023). Still, its effect on WLB has been investigated less. In our study, we hypothesize that sexual harassment and workplace violence would likely reduce WLB. Sexual harassment and workplace violence are dangerous not only to the health and well-being of employees but also to organizational return on investment, signifying organizational health and well-being (Vara-Horna et al., 2023; Willness et al., 2007).

Given the vital role of WLB in employee retention and organizational success, understanding the factors that disrupt this balance is crucial (Lazǎr et al., 2010; Manna et al., 2023; Salehi, 2007). While extensive research has examined work-family balance and general work-health considerations, the association of workplace psychosocial hazards—particularly conflicts, sexual harassment, workplace violence, and rivalry—with WLB remains underexplored.

This study aims to address this research gap by:

1. Evaluating the extent to which workplace psychosocial hazards—conflicts (status similar and status dissimilar), sexual harassment, workplace violence, and rivalry—are associated with employees’ WLB;
2. Evaluating the varying degree to which WLB differs among demographic and employee groups;
3. Based on findings, suggesting the potential practical implications for organizations in fostering a supportive psychosocial environment that enhances WLB.

## 2 Materials and methods

### 2.1 Study population

A cross-sectional study design was used to evaluate the association between the selected workplace psychosocial hazards (conflicts, rivalry, psychological or physical abuse, and sexual harassment) and WLB. This study utilized data from the national workforce survey “Work Conditions and Risks in Latvia 2019–2021,” which aims to evaluate occupational health and safety factors, including selected workplace psychosocial hazards.

The initial population of the survey “Work Conditions and Risks in Latvia 2019–2021” was representative of Latvia’s working-age population. However, for a more homogeneous study population, our analysis focused on paid employees, excluding other surveyed respondents, such as the self-employed and those on maternity leave.

Respondents were randomly selected from various regions and industries. The average age of the respondents (*n* = 2,471) was 44.7 ± 13.7 years (ranging from 18 to 74 years), with 40.9%, *n* = 1,010 male and 59.1%, *n* = 1,461 female. Data was collected through computer-assisted personal interviewing (CAPI) at the respondents’ residences.

### 2.2 Study variables

The outcome variable in this study was WLB, measured by the question: “How often do the following situations occur in your workplace: Lack of balance between work and private life?” The possible responses were: “rather often,” “sometimes,” “rarely,” “never,” and “hard to say.” The responses “rather often,” “sometimes,” and “rarely” were combined into one group, which is those who lack WLB. Respondents who reported never experiencing a lack of WLB were used as the reference group, while those who selected “hard to say” were excluded from the analysis, as their responses did not provide clear insight into their experiences or opinions on the assessed variable.

Selected workplace psychosocial hazards (independent variables) concerning WLB were examined. These factors were assessed using the question: “How often do the following situations occur in your workplace: Conflicts with managers; Individual conflicts between co-workers; Conflicts between worker groups; Conflicts with clients; Rivalry between co-workers; Physical abuse or threats; Psychological abuse or threats; Sexual harassment?.” Responses included: “rather often,” “sometimes,” “rarely,” “never,” and “hard to say.” Also, in this case respondents who chose “hard to say” were excluded from the analysis.as their responses did not offer definitive information about their experiences or perspectives on the particular situation. Data were categorized and analyzed as dichotomous variables. Respondents who selected “rather often,” “sometimes,” or “rarely” were grouped into the exposed category, while those who answered “never” were used as the reference group.

Various work characteristics were analyzed, such as job position (head of the company, senior or middle manager, senior or intermediate level specialist, service and sales employee, skilled or unskilled workers), work sector (public or private), company size (number of workers 1–10, 11–49, 50–249, 250 workers and more), length of service (less than 1 year, 1–5 years, 5–10 years, 10 years or more), and remote or on-site work.

Gender, age, and education were included in the regression models, and the odds ratios were adjusted for these factors. Age was categorized as follows: 18–24, 25–34, 35–44, 45–54, and 55–74. Education levels were classified as primary school or elementary education, secondary or vocational school education, and higher education. We used income quartiles to analyze income distribution within the population by dividing the dataset into four equal parts, each representing 25% of the population.

### 2.3 Data statistical analysis

The association between the selected workplace psychosocial hazards and WLB was analyzed by using binomial logistic regression, with results expressed as odds ratios (ORs) and 95% confidence intervals (CIs), adjusted for gender, age, and education (aOR). To assess multicollinearity among gender, age, and education, the Spearman correlation coefficient was calculated, revealing no significant multicollinearity. The analysis was conducted with IBM SPSS Statistics 29 (IBM Corporation, Armonk, New York, NY, USA).

### 2.4 Ethical considerations and data protection

This study was conducted according to the guidelines of the Declaration of Helsinki and approved by the Institutional Review Board—Ethics Committee of Rīga Stradiņš University (protocol No. 22–2/601/2021, 22 December 2021). The study obtained verbal informed consent from all participants before data collection, ensuring they were fully aware of the study’s purpose and their voluntary participation. Strict data protection measures were implemented, including anonymizing responses to prevent the identification of individual participants and securely storing all data in compliance with the approved ethical guidelines.

## 3 Results

The study revealed significant associations between workplace psychosocial hazards and WLB, highlighting the pervasive impact of conflicts, abuse, and harassment on employees’ well-being. These findings underscore the importance of addressing psychosocial hazards to foster healthier and more balanced work environments.

A lack of WLB is prevalent among Latvian employees, with every third respondent (30.9%, *n* = 762) reporting it; part of these respondents reported a lack of WLB “rather often” (4.7%, *n* = 117), and 12.5% (*n* = 308) reported it as “occasional.” As detailed in Table 1, the odds of experiencing WLB imbalance are slightly higher for males (aOR = 1.28, 95% CI 1.07–1.54, *p* < 0.01) after adjustment for age and education.

**Table 1:** The odds of lack of WLB in association with sociodemographic factors.

	Distribution, *n* (%)	WLB, *n* (%)	WLB, OR (CI 95%)^a^, Not adjusted	WLB, aOR (CI 95%)^a^, Adjusted^b^
Gender	2,471	763		
male	1,010 (40.9)	332 (43.5)	1.17 (0.98–1.39)	1.28 (1.07–1.54)**
female	1,461 (59.1)	431 (56.5)	1	1
Age	2,470	763		
18–24 years	180 (7.3)	73 (9.6)	2.29 (1.62–3.23)*	2.71 (1.89–3.88)*
25–34 years	519 (21.0)	192 (25.1)	1.97 (1.54–2.53)*	1.82 (1.41–2.35)*
35–44 years	510 (20.6)	180 (23.6)	1.84 (1.43–2.36)*	1.71 (1.32–2.22)*
45–54 years	541 (21.9)	153 (20.1)	1.33 (1.03–1.71)***	1.29 (1.00–1.68)
55–74 years	720 (29.2)	165 (21.6)	1	1
Education	2,469	762		
Primary school or elementary education	163 (6.6)	20 (2.6)	0.19 (0.12–0.32)*	0.15 (0.09–0.25)*
Secondary or vocational school education	1,355 (54.9)	350 (45.9)	0.51 (0.39–0.66)*	0.49 (0.41–0.59)*
Higher education	951 (38.5)	392 (51.5)	1	1
Salary	2,255	700		
1st quartile (lowest)	560 (24.8)	111 (15.9)	0.30 (0.23–0.39)*	0.46 (0.34–0.62)*
2nd quartile	534 (23.7)	151 (21.6)	0.48 (0.37–0.62)*	0.65 (0.49–0.86)**
3rd quartile	624 (27.7)	195 (27.9)	0.55 (0.43–0.70)*	0.63 (0.49–0.81)*
4th quartile (highest)	537 (23.8)	243 (34.6)	1	1

^*^p < 0.001; **p < 0.01; ***p < 0.05.

a The reference category for the WLB group is the group of respondents who did not have a lack of balance between work and private life at work (answered “never”).

b Adjusted for gender, age, and education.

WLB challenges also vary significantly across age groups. Younger respondents face the greatest odds of imbalance, with the 18–24 age group having two times higher odds (aOR = 2.71, 95% CI 1.89–3.88, *p* < 0.001) than the oldest age group (55–74 years). The trend indicates that the likelihood of experiencing WLB imbalance decreases with age (Table 1).

Educational attainment plays a crucial role. Respondents with lower education levels—such as primary or elementary education—demonstrate significantly reduced odds of WLB imbalance (aOR = 0.15, 95% CI 0.09–0.25, *p* < 0.001) compared to those with higher education. Similarly, income levels influence WLB outcomes. Respondents in the lowest income quartile show significantly lower odds (aOR = 0.46, 95% CI 0.34–0.62, *p* < 0.001) of reporting a lack of WLB compared to those in the highest quartile (Table 1).

Table 2 examines the associations between workplace psychosocial hazards and WLB. The findings highlight several psychosocial risks contributing to WLB imbalance. Among workplace psychosocial hazards, sexual harassment is most strongly associated with WLB imbalance. Employees reporting incidents of sexual harassment had significantly higher odds of experiencing WLB difficulties (aOR = 4.90, 95% CI 2.06–11.67, *p* < 0.001) compared to those who did not report such incidents.

**Table 2:** The odds of lack of WLB in association with selected workplace psychosocial hazards.

	Distribution, *n* (%)	WLB, *n* (%)	WLB, OR (CI 95%)^a^, Not adjusted	WLB, aOR (CI 95%)^a^, Adjusted^b^
Sexual harassment	2,459	755		
Yes	26 (1.1)	18 (2.4)	5.43 (2.34–12.62)*	4.90 (2.06–11.67)*
No	2,433 (98.9)	737 (97.6)	1	1
Psychological abuse or threats	2,454	755		
Yes	392 (16.0)	223 (29.5)	3.80 (3.04–4.75)*	3.47 (2.75–4.35)*
No	2062 (84.0)	532 (70.5)	1	1
Individual conflicts between co-workers	2,438	753		
Yes	1,047 (42.9)	490 (65.1)	3.77 (3.15–5.52)*	3.40 (2.82–4.10)*
No	1,391 (57.1)	253 (34.9)	1	1
Conflicts between worker groups	2,367	724		
Yes	627 (26.5)	330 (45.6)	3.80 (3.13–4.61)*	3.35 (2.74–4.12)*
No	1740 (73.5)	394 (54.4)	1	1
Conflicts with managers	2,402	739		
Yes	1,192 (49.6)	519 (70.2)	3.48 (2.88–4.18)*	3.23 (2.66–3.91)*
No	1,210 (50.4)	220 (29.8)	1	1
Rivalry between co-workers	2,411	743		
Yes	758 (31.4)	384 (51.7)	3.70 (3.08–4.45)*	3.10 (2.56–3.75)*
No	1,653 (68.6)	359 (48.3)	1	1
Conflicts with clients	2,366	730		
Yes	1,093 (46.2)	469 (64.2)	2.91 (2.43–3.49)*	2.58 (2.13–3.12)*
No	1,273 (53.8)	261 (35.8)	1	1
Physical abuse or threats	2,461	758		
Yes	156 (6.3)	79 (10.4)	2.46 (1.78–3.41)*	2.24 (1.60–3.14)*
No	2,305 (93.7)	679 (89.6)	1	1

^*^p < 0.001; **p < 0.01; ***p < 0.05.

a The reference category for the WLB group is the group of respondents who did not have a lack of balance between work and private life at work (answered “never”).

b Adjusted for gender, age, and education.

Employees subjected to psychological abuse or threats also faced elevated odds of WLB imbalance (aOR = 3.47, 95% CI 2.75–4.35, *p* < 0.001). These hazards represent a significant barrier to achieving a balanced work-life dynamic.

The findings in Table 2 also emphasize the substantial impact of conflicts on WLB. Individual conflicts between co-workers are particularly problematic, increasing the odds of imbalance (aOR = 3.40, 95% CI 2.82–4.10, *p* < 0.001). Similarly, conflicts between worker groups contribute significantly to WLB challenges (aOR = 3.35, 95% CI 2.74–4.12, *p* < 0.001). Employees experiencing conflicts with their managers had significantly increased odds of WLB imbalance (aOR = 3.23, 95% CI 2.66–3.91, *p* < 0.001). This underscores the critical role of leadership in mitigating workplace psychosocial risks.

Rivalry between co-workers (aOR = 3.10, 95% CI 2.56–3.75, *p* < 0.001) and conflicts with clients (aOR = 2.58, 95% CI 2.13–3.12, *p* < 0.001) further exacerbate the risk of WLB imbalance. Finally, physical abuse and threats were associated with a modest but significant increase in WLB imbalance (aOR = 2.24, 95% CI 1.60–3.14, *p* < 0.001), as seen in Table 2.

Beyond workplace conflicts, various work characteristics were also linked to WLB imbalance. Our findings indicate WLB differences depending on various work characteristics, such as job position, work sector, company size, length of service, and remote or on-site work (Table 3). A strong association was found between job roles and WLB imbalance. Respondents in managerial positions demonstrated the highest odds of WLB challenges. Heads of companies were at significantly increased risk (aOR = 4.21, 95% CI 1.93–9.18, *p* < 0.001) compared to unskilled workers who served as a reference group. Similarly, senior managers and middle managers showed higher odds (aOR = 3.13, 95% CI 1.93–5.08, *p* < 0.001), followed by senior-level specialists (aOR = 3.08, 95% CI 1.91–4.96, *p* < 0.001). Even intermediate-level specialists exhibited increased odds of WLB imbalance (aOR = 2.23, 95% CI 1.42–3.51, *p* < 0.001), suggesting that higher responsibility roles may contribute to greater WLB challenges.

**Table 3:** The odds of lack of WLB in association with work characteristics.

	Distribution, *n* (%)	WLB, *n* (%)	WLB, OR (CI 95%)^a^, Not adjusted	WLB, aOR (CI 95%)^a^, Adjusted^b^
Job category	2,469	762		
Head of the company	36 (1.5)	19 (2.5)	7.01 (3.301–14.90)*	4.21 (1.93–9.18)*
Senior manager or middle manager	245 (9.9)	108 (14.2)	5.10 (3.25–7.99)*	3.13 (1.93–5.08)*
Senior level specialist	393 (15.9)	176 (23.1)	5.24 (3.44–7.99)*	3.08 (1.91–4.96)*
Intermediate level specialist	392 (15.9)	139 (18.2)	3.54 (2.31–5.41)*	2.23 (1.42–3.51)*
Service and sales employee	511 (20.7)	128 (16.8)	2.16 (1.41–3.29)*	1.58 (1.02–2.44)***
Skilled worker	654 (26.5)	160 (21.0)	2.09 (1.38–3.16)*	1.57 (1.03–2.41)***
Unskilled worker	238 (9.6)	32 (4.2)	1	1
Primary work sector	2,409	744		
Public sector	906 (37.6)	307 (41.3)	1.25 (1.05–1.49)***	1.21 (1.00–1.48)
Private sector	1,503 (62.4)	437 (58.7)	1	1
Number of workers	2,268	727		
1–10 workers	559 (24.6)	129 (17.7)	0.41 (0.32–0.54)*	0.53 (0.40–0.70)*
11–49 workers	662 (29.3)	189 (26.0)	0.55 (0.43–0.71)*	0.63 (0.49–0.82)*
50–249 workers	586 (25.8)	215 (29.6)	0.80 (0.62–1.03)	0.90 (0.69–1.16)
250 workers or more	461 (20.3)	194 (26.7)	1	1
Length of service	2,466	760		
Less than 1 year	434 (17.6)	108 (14.2)	0.70 (0.54–0.91)**	0.50 (0.37–0.69)*
1–5 years	820 (33.3)	258 (33.9)	0.96 (0.78–1.19)	0.72 (0.56–0.91)**
5–10 years	422 (17.1)	139 (18.3)	1.03 (0.80–1.33)	0.86 (0.66–1.13)
10 years or more	790 (32.0)	255 (33.6)	1	1
Working remotely	2,434	753		
Yes	626 (25.7)	301 (40.0)	2.79 (2.30–3.36)*	2.12 (1.70–2.64)*
No	1808 (74.3)	452 (60.0)	1	1

^*^p < 0.001; **p < 0.01; ***p < 0.05.

a The reference category for the WLB group is the group of respondents who did not have a lack of balance between work and private life at work (answered “never”).

b Adjusted for gender, age, and education.

The data in Table 3 also suggest that employees in larger companies (250+ workers) who served as a reference group, are more likely to experience WLB imbalance compared to those in smaller organizations (1–10 workers) (aOR = 0.53, 95% CI 0.40–0.70, *p* < 0.001) and 11–49 workers (aOR = 0.63, 95% CI 0.49–0.82, *p* < 0.001). Less experienced workers—with length of service less than 1 year (aOR = 0.50, 95% CI 0.37–0.69, *p* < 0.001) and 1 to 5 years (aOR = 0.72, 95% CI 0.56–0.91, *p* < 0.01) demonstrate a lack of WLB less compared to respondents whose length of service is 10 years and more who served as a reference group. Lastly, employees working remotely had significantly greater odds of WLB imbalance (aOR = 2.12, 95% CI 1.70–2.64, *p* < 0.001) compared to those working on-site, reinforcing the impact of work modality on WLB.

Heads of the companies (aOR = 4.21, 95% CI 1.93–9.18), managers (aOR = 3.13, 95% CI 1.93–5.08), and specialists (senior level aOR = 3.08, 95% CI 1.91–4.96 and intermediate level aOR = 2.23, 95% CI 1.42–3.51) more often face a lack of WLB compared to unskilled workers, and respondents whose length of service is 10 years or more (compared with less experienced workers).

## 4 Discussion

This discussion explores the significant associations identified between workplace psychosocial hazards and WLB, emphasizing the role of conflicts, harassment, and leadership quality in shaping employee well-being within the Latvian workforce.

### 4.1 Workplace psychological abuse and threats

Psychological abuse and threats in their negative association with WLB are triple significant (aOR = 3.47) after adjustment for age and gender. Thus, our results support the findings indicating the detrimental effect of workplace psychosocial hazards on the WLB of employees (Leka & Jain, 2018). In addition, they demonstrate how employees rank these conflict-inducing workplace psychosocial hazards concerning their impact on their WLB.

Furthermore, psychological abuse and threats are leading the association with negative WLB, confirming that a majority of violent behavior in workplaces is psychological compared to physical abuse and threats (Sui et al., 2019; Maes et al., 2000; Sroka & Vveinhardt, 2020). The prominent association of psychological violence and threats with negative WLB in our results agrees with a study evaluating the impact of psychological violence and threats on WLB (Gorenak & Popovic, 2014). It identified not only the adverse effects of psychosocial violence and threats on employees psychologically but on their physical health, too (Gorenak & Popovic, 2014). Furthermore, psychological violence damages the psychosocial capital of employees, which includes interpersonal and inter-group relationships and trust (Luthans & Youssef, 2004; Shahidi et al., 2021). The quality of leadership and the organizational climate determine the successful resolution of violated or compromised psychosocial capital, including trust (Shahidi et al., 2021).

Moreover, leadership style predicts conflict management style in the workplace (Naqvi & Anjum, 2024). Thus, how leadership sets an example in conflict management is vital in improving employee WLB (Susanne & Claudia, 2016). Therefore, to improve the quality of the psychosocial work environment, it would be best to start with improving the leadership quality (Susanne & Claudia, 2016; Wood et al., 2019). Such an approach is consistent with a ‘hierarchy of controls’ for the improving psychosocial environment at work (Shahidi et al., 2021; Dollard et al., 2007; LaMontagne et al., 2007). It is because of leadership’s broad influence on organizational processes (Wood et al., 2019; Härenstam, 2008). Furthermore, quality leadership tends to influence various processes simultaneously because many factors are intertwined in a psychosocial environment (Shahidi et al., 2021). Thus, the example of leadership has a powerful effect on employees (Rugulies, 2019; Härenstam, 2008; Berthelsen et al., 2018).

Our results demonstrate how closely conflicts follow psychological violence and threats (aOR = 3.47). Furthermore, the highest ranked in their negative association with WLB are conflicts that can be powerfully managed by leaders—such as conflicts between co-workers (aOR = 3.40) and conflicts between worker groups (aOR = 3.35). For example, conflicts between co-workers and worker groups influence WLB negatively more than conflicts with clients (aOR = 2.58). Thus, in our data, interpersonal conflicts and conflicts between worker groups appear to affect employee WLB the most. Conflicts with managers (aOR = 3.23) impact WLB less than conflicts with co-workers or between worker groups. However, managers may not proactively set a good enough example to prevent inter-group conflicts. For example, managers may not be equipped to deal with workplace psychosocial hazards that thrive or manage conflicts in such a way that they have a positive rather than negative impact on employees.

Workplace affects WLB primarily through conflicts at work identified by regulatory agencies a decade ago (European Agency for Safety and Health at Work, 2012). However, evidence-based data to substantiate this assertion is hard to find. One of the reasons for this is that well-being has been used as a proxy for WLB in most research dealing with how various workplace psychosocial hazards influence employee life outside work (Moreira et al., 2023; Wang, 2022; Andrade & Lousã, 2021). Another reason is assuming that the psychological component of well-being exists outside of work and promotes work accomplishments instead of being an integral part of the work environment (Moreira et al., 2023). Our data supports the opposite. It shows that psychosocial abuse and threats are leading psychosocial hazards in decreased WLB of employees. Thus, a decrease in the potential of psychological abuse and threats should be a priority area for the leadership of organizations in enhancing employee WLB. This finding aligns with studies demonstrating that working attitudes and characteristics related to the manager and colleagues affect WLB significantly (Wong & Chan, 2021).

Further in our discussion, we demonstrate how a salary increase is associated with decreased WLB. First, it shows the decrease of WLB with increased seniority in career positions, as indicated by the increase in salary. However, it also shows the prominence of psychosocial well-being compared to salary in its positive effect on WLB.

Because of the significance of psychosocial hazards, especially psychological violence and threats, and conflicts on WLB, our results suggest that promoting conflict resolution and psychosocial well-being creation skills at the managerial level would be the best approach to improving these areas for employees (Martin et al., 2016). Furthermore, such an approach aligns with the organizational duties of leaders, where they are more involved in interpersonal challenges due to being responsible for people development and dealing with conflicts (Bonsaksen et al., 2019).

### 4.2 Physical abuse, threats, conflicts with clients, rivalry at work

Physical abuse, threats, and conflicts with clients, in comparison to psychological abuse and threats and conflicts, are the least associated with negative WLB. Their short-term, momentary nature lends itself to resolution on a case-by-case basis and makes it easier to deal with (Sackett & Saunders, 1999). As a result, they may not have a lasting effect on WLB compared to psychological violence and threats, for example, which are more pervasive.

Rivalrous behavior has been investigated in the context of conflict, motivation, unethical behaviors, and work performance outcomes. Still, there is scant evidence-based data about rivalry and WLB (Kilduff, 2014; Kilduff & Galinsky, 2017; To et al., 2018; De Clercq et al., 2022). Therefore, we included a question on rivalry in our survey to explore how much it threatens WLB. We found an association of rivalry with negative WLB (aOR = 3.10). The magnitude of this association places rivalry within the conflict cluster (after individual conflicts with coworkers, conflicts with managers, and conflicts between worker groups but before conflicts with clients). Rivalry links to conflict through having a component of maximizing one’s gains over those of others, while cooperativeness, in contrast, seeks to equalize these gains mutually (Houston et al., 2000). Thus, rivalry can lead to conflict. In addition, rivalry significantly decreases well-being because it leads to the transfer of resources (such as time and energy) from the life domain to the work domain of WLB (Regina & Allen, 2023).

### 4.3 Workplace sexual abuse and harassment

In addition, we found that workplace sexual abuse and harassment are associated with negative WLB the most, retaining five-fold significance after adjustment for age and gender (aOR = 4.90). It supersedes all other psychosocial hazards in its association with negative WLB. However, the results are based only on 26 respondents out of 2,459. Comparisons to other studies are challenging because there are limited investigations explicitly linking workplace sexual harassment to WLB. Nevertheless, our findings align with prior research emphasizing the pervasive impact of sexual harassment in organizational settings.

The prominence of workplace sexual harassment in its association with negative WLB may be explained by its tendency to permeate the work environment, similar to psychological abuse and threats (Willness et al., 2007; Lapierre et al., 2005). Therefore, workplace sexual harassment has a detrimental effect on mental health and job satisfaction globally, which are integral components of broader WLB (Liang, 2024). Although the previously mentioned meta-analysis evaluated job satisfaction, this narrower focus does not fully capture the multidimensional nature of WLB, which includes both work and non-work-life components. Our study, therefore, extends this understanding by demonstrating the significant potential effect of workplace sexual harassment on broader dimensions of WLB.

The emotional turmoil, stress, and mental health problems caused by sexual harassment can severely disrupt an individual’s personal and professional life (Chang, 2024). It is because patterns of workplace sexual harassment often manifest as systemic issues, which lead to persistent psychological distress and erode trust within organization (Karami et al., 2024). Experiencing sexual harassment not only affects victims’ ability to maintain WLB but also reduces their desire to return to work (Chen et al., 2023). Furthermore, when organizations fail to adequately address sexual harassment, victims often perceive this as secondary harm, exacerbating feelings of betrayal and disengagement (Willness et al., 2007; Adams & Bray, 1992; Fitness, 2000).

Sexual harassment also has organizational consequences (Raihan et al., 2020). Its resultant conflicts lead to decreased productivity (by 43.1%) and increased turnover intentions (by 15.2%) among victims (Vara-Horna et al., 2023). Alarmingly, similar negative impacts are observed in witnesses, with corresponding values of 39.6% for decreased productivity and 11.3% for increased turnover intentions (Vara-Horna et al., 2023). These findings emphasize the far-reaching implications of sexual harassment, not only for individuals directly involved but also for the broader workplace environment.

Despite workplace sexual harassment being one of the most damaging and ubiquitous barriers to career success and satisfaction for women, as identified by a recent meta-analysis, its effects on WLB remain underexplored (Matisāne et al., 2024). Considering its well-documented consequences on job satisfaction, it is imperative to investigate its broader implications for WLB (Liang, 2024). Our study provides preliminary evidence in this area, highlighting the necessity for organizational interventions.

The significant association of workplace sexual harassment with negative WLB underscores the importance of addressing this issue through robust anti-harassment policies, effective reporting mechanisms, and cultural change. The interplay of power dynamics and cultural factors perpetuates harassment in workplaces, necessitating comprehensive strategies to counteract these influences (Raihan et al., 2020). Proactive leadership and organizational accountability are essential in mitigating the long-term impacts of sexual harassment on both WLB and workplace well-being. This evidence iterates the previously discussed significance (concerning psychological abuse and threats and conflicts) of organizational leadership quality, which is highly dependent on the setup of organizational values.

### 4.4 Effects in different demographic and employee groups

When identifying the detrimental effect of workplace psychosocial hazards—the environment where such workplace psychosocial hazards take hold and thrive points to a possibility of the need for more intentional and strategic leadership to eliminate them; the role of supervisors and leadership is to set the example, create an environment where harmful workplace psychosocial hazards cannot thrive, and systematically continue re-evaluation and addressing the health of the psychosocial environment at the workplace (Bailyn, 2011). However, according to our results, leadership/senior management is affected the most by negative WLB.

Our findings agree with studies identifying that older age group of workers are most satisfied with their WLB (Ministry of Social Development, 2016), probably due to their ability to place work-non-work boundaries and sustain WLB (Richert-Kaźmierska & Stankiewicz, 2016). Younger workers are more likely to report the occurrence of negative WLB due to parental responsibilities and the initial phase of career development (Ministry of Social Development, 2016).

After adjustment for age and education, the lack of WLB becomes significant for males, which is inconsistent with the data of the studies that reveal work-life imbalance is more prevalent in the female working population (van & Lippényi, 2020). Our explanation could be the difference in gender and work-related attitudes—female employees have more positive work-related attitudes toward the organization, a higher level of commitment, and job satisfaction than men (Karkoulian et al., 2016), which affects the subjective perception of WLB.

Regarding various work characteristics, our results demonstrate worse WLB with higher employee rank, education, salary, and years of experience. These can be explained through increased workload as a mediating factor—the greater the workload employees have to overcome, the more affected WLB is (Natanael et al., 2023). Poor WLB in higher employee ranks is in line with studies that highlight leaders’ unique challenges and perceptions (Hambrick et al., 2005; Brue, 2018). Senior staff members engage in more complex planning, decision-making, and higher-risk issues, leading to increased stress (Bonsaksen et al., 2019; Pangert & Schüpbach, 2013; Ten Brummelhuis et al., 2014). This inference can also be applied to employees with a higher salary, education level or years of experience. Employees with higher education demonstrate lower WLB due to higher demands and responsibility in their jobs, which leads to an increased risk of burnout (Shanafelt et al., 2012; Kang et al., 2020). Likewise, higher-ranking employees are expected to be constantly available and tend to work longer hours, even when unwell (Pangert & Schüpbach, 2013; Ten Brummelhuis et al., 2014; Dietz et al., 2020). In contrast to studies that suggest negative WLB is a universal issue, our results show that WLB worsens with higher employee rank (Humphries et al., 2020).

Regarding salary and WLB, very few studies have been performed that we could use to compare and contrast our data. However, we identified a recent meta-analysis that demonstrates the limited value of an individual’s salary in enhancing subjective well-being (the term often used in WLB research instead of WLB), i.e., happiness (Steel et al., 2018). Equally, decades of happiness research have provided evidence of salary’s limited effect on an individual’s happiness (Helliwell & Putnam, 2004; Helliwell et al., 2022). Furthermore, incentivizing employees with extreme wages leads to a reverse effect, especially in terms of creativity and performance (Ramm et al., 2021; Charness & Grieco, 2019).

WLB decreases with the increase of years of experience at work. After adjustment for age, gender, and education, WLB decreases consistently through 1 to more than 10 years of service. The best WLB is for employees working less than a year, followed by 1–5 years. The negative impact of years of service on WLB is a pressing issue that needs to be addressed. Possible explanations for the worsening of WLB with years of experience in our results could be less workload for employees who have started their jobs, a period of training and onboarding followed by taking on less responsibility in the first few years of work. We could draw on limited research on years of service and WLB. Indirect evidence for the negative impact of years of service on WLB comes from studies demonstrating increased job demands, lack of replacement possibilities, stress, burnout, and health afflictions leading to poor WLB (Shanafelt et al., 2012; Lott & Wöhrmann, 2023; Nordenmark et al., 2012).

Remote work is associated with higher odds of negative WLB due to increased effort demands (Felstead & Henseke, 2017) and after-hours work communication, both hallmarks of the technology-enabled work environment (Kim & Chon, 2022). To mitigate these challenges, organizations must adopt (Matisāne et al., 2024; Felstead & Henseke, 2017; Walsh et al., 2024) an employee-centric approach, emphasizing flexibility, agility and interpersonal support (Gierlich-Joas, 2021). Interpersonal relationships are essential for improving remote employees’ WLB and well-being, as disruptions to these relationships or increases in workplace psychosocial hazards significantly impact WLB negatively (Buonomo et al., 2024; Brunelle & Fortin, 2021).

### 4.5 Limitations

Our study has several limitations that warrant consideration, including cross-sectional design, potential reverse causality, focus on specific WLB dimensions, sample size for sexual harassment, lack of detailed information on conflicts (e.g., frequency, length etc.), self-reported data, and lack of data on additional confounders.

The cross-sectional nature of our study prevents causal inferences, as exposure and outcome are measured simultaneously (Savitz & Wellenius, 2023). This design may lead to exposure misclassification, where an etiological process is linked to an outcome inaccurately (Savitz, 2009). While this limitation affects causal interpretations, cross-sectional designs remain valuable for evaluating chronic or episodic conditions such as conflicts, sexual harassment, and rivalry (Hudson et al., 2005). Our findings provide meaningful associations that can guide future longitudinal-study research.

Another limitation is the potential for reverse causality. For example, prolonged illness may affect WLB and lead to conflicts that did not directly originate from the work environment (De Dreu et al., 2004). Future research could address this issue by exploring additional variables such as family commitments, health conditions, overtime, absenteeism, presenteeism, and available workplace support. These factors may offer a more nuanced understanding of WLB dynamics.

Cross-sectional studies often focus narrowly on one dimension of WLB (Weziak-Bialowolska et al., 2020). Our study prioritized specific domains—such as psychological threats, physical threats, conflict, rivalry, and sexual harassment—that are most detrimental to WLB. While this approach highlights key psychosocial hazards, it does not address broader family-to-work conflicts, which were beyond the scope of this study. Future research could investigate the mediation effects of family-to-work conflicts on WLB.

Another limitation of this study is the lack of detailed information regarding the intensity and duration of workplace conflicts. The data did not differentiate between one-time events and ongoing or repeated conflicts, which could have varying impacts on WLB. Future research should consider incorporating measures to capture the frequency, persistence, and severity of conflicts to provide a more nuanced understanding of their effects on WLB. In addition, we would like to emphasize that respondents who selected “hard to say” were excluded from the analysis, as their responses did not provide clear insight into their experiences; however, these answers may indicate workplace ambiguity or lack of awareness, which are important aspects of psychosocial hazards and warrant further exploration in future studies.

The sample of respondents reporting workplace sexual harassment was small (*n* = 26), limiting the generalizability of our findings. However, these preliminary results provide important insights into the significant impact of sexual harassment on WLB, encouraging further exploration in this under-researched area.

Our study relied on self-reported WLB, which is subject to subjective interpretation. However, historical evidence supports the validity of self-reported data in participatory research (Faller, 2021). Moreover, studies show that self-reported mental health outcomes strongly align with identified psychosocial environment profiles, supporting the reliability of our data (Shahidi et al., 2021; Pawlicka et al., 2020; Bouzari & Karatepe, 2020).

The OR were adjusted for key confounders, including gender, age, and education; however, incorporating additional variables such as personality traits or family responsibilities could have enriched the analysis. Furthermore, exploring the potential influence of family dynamics, individual coping mechanisms, and personal resilience may offer a more comprehensive understanding of the findings. These aspects were not included in our study but represent valuable directions for future research.

### 4.6 Practical implications

Despite the above-mentioned limitations, our data demonstrates preliminary causal evidence based on the strength of the associations we established (Savitz & Wellenius, 2023). It affirms that the psychosocial setting where employees work is a significant source of support and stress (Savitz & Wellenius, 2023). The findings of this study underline critical areas for organizational action to improve WLB among employees. First, addressing psychosocial hazards, such as workplace conflicts, psychological abuse, and sexual harassment, is paramount. Organizations should prioritize implementing robust conflict resolution mechanisms and anti-harassment policies, ensuring a safer and more supportive workplace environment. Leadership development emerges as a vital strategy, given the significant role managerial conflicts play in WLB. Training managers to foster inclusive leadership styles and effectively mediate disputes can help mitigate WLB challenges associated with interpersonal and inter-group conflicts. Organizations may also benefit from adopting a top-down approach to model positive workplace behaviors. The results also suggest that organizations should consider customized support systems for employees in roles with heightened responsibility (e.g., senior managers and specialists), as they exhibit a higher likelihood of WLB imbalance. Practical measures may include workload redistribution, flexible scheduling, and regular well-being assessments. Finally, the challenges associated with remote work require targeted strategies, such as clear boundaries for after-hours communication and proactive engagement initiatives to reduce isolation. Organizations should establish structured yet flexible remote work policies that foster connectivity and productivity without sacrificing employee well-being.

By addressing these areas, organizations can not only enhance employee satisfaction but also drive productivity and long-term retention, creating a win-win environment for both employees and employers.

## 5 Conclusion

To our knowledge, this is the first study to estimate the association between WLB, psychological and physical abuse and threats, conflicts, rivalry, and workplace sexual harassment. Our study demonstrates a strong association of all types of workplace conflicts, abuse, and harassment with poor WLB. It suggests the necessity of strong leadership to manage different workplace conflicts and create a positive psychosocial work environment.

## Data Availability

Publicly available datasets were analyzed in this study. This data can be found at: https://dataverse.rsu.lv/dataset.xhtml?persistentId=doi:10.48510/FK2/QIX7L1 RSU Dataverse repository.
